# Machine learning-based quantification for disease uncertainty increases the statistical power of genetic association studies

**DOI:** 10.1093/bioinformatics/btad534

**Published:** 2023-09-04

**Authors:** Jun Young Park, Jang Jae Lee, Younghwa Lee, Dongsoo Lee, Jungsoo Gim, Lindsay Farrer, Kun Ho Lee, Sungho Won

**Affiliations:** Department of Public Health Sciences, Graduate School of Public Health, Seoul National University, Seoul 08826, Korea; Neurozen Inc., Seoul 06168, Korea; Gwangju Alzheimer’s & Related Dementia Cohort Research Center, Chosun University, Gwangju 61452, Korea; Gwangju Alzheimer’s & Related Dementia Cohort Research Center, Chosun University, Gwangju 61452, Korea; Department of Public Health Sciences, Graduate School of Public Health, Seoul National University, Seoul 08826, Korea; Department of Public Health Sciences, Graduate School of Public Health, Seoul National University, Seoul 08826, Korea; Gwangju Alzheimer’s & Related Dementia Cohort Research Center, Chosun University, Gwangju 61452, Korea; Department of Biomedical Science, Chosun University, Gwangju 61452, Korea; Departments of Medicine (Biomedical Genetics), Neurology, and Ophthalmology, Boston University Chobanian & Avedisian School of Medicine, Boston, MA 02118, United States; Departments of Epidemiology and Biostatistics, Boston University School of Public Health, Boston, MA 02118, United States; Gwangju Alzheimer’s & Related Dementia Cohort Research Center, Chosun University, Gwangju 61452, Korea; Department of Biomedical Science, Chosun University, Gwangju 61452, Korea; Korea Brain Research Institute, Daegu 41068, Korea; Department of Public Health Sciences, Graduate School of Public Health, Seoul National University, Seoul 08826, Korea; Interdisciplinary Program in Bioinformatics, Seoul National University, Seoul 08826, Korea; Institute of Health and Environment, Seoul National University, Seoul 08826, Korea; RexSoft Inc, Seoul 08826, Korea

## Abstract

**Motivation:**

Allowance for increasingly large samples is a key to identify the association of genetic variants with Alzheimer’s disease (AD) in genome-wide association studies (GWAS). Accordingly, we aimed to develop a method that incorporates patients with mild cognitive impairment and unknown cognitive status in GWAS using a machine learning-based AD prediction model.

**Results:**

Simulation analyses showed that weighting imputed phenotypes method increased the statistical power compared to ordinary logistic regression using only AD cases and controls. Applied to real-world data, the penalized logistic method had the highest AUC (0.96) for AD prediction and weighting imputed phenotypes method performed well in terms of power. We identified an association (*P*<$5.0\times 10^{-8}$) of AD with several variants in the *APOE* region and rs143625563 in *LMX1A*. Our method, which allows the inclusion of individuals with mild cognitive impairment, improves the statistical power of GWAS for AD. We discovered a novel association with *LMX1A*.

**Availability and implementation:**

Simulation codes can be accessed at https://github.com/Junkkkk/wGEE_GWAS.

## 1 Introduction

Alzheimer’s disease (AD), the most common cause of dementia, is a multifactorial condition influenced by innate and lifestyle risk factors (2022). The disease is highly heritable, with an estimated 58%–79% (*h*^2^) of the liability explained by genetic factors (Gatz *et al.* 2006), most notably the *APOE* genotype and more than 75 other loci identified by genome-wide association studies (GWAS) conducted in European, African, and Asian ancestry cohorts (Kang *et al.* 2021, Kunkle *et al.* 2021, Bellenguez *et al.* 2022, Sherva *et al.* 2022).

Despite the success of AD GWAS conducted in large cohorts of AD cases and controls of European ancestry, the challenge of assembling sufficiently large well characterized cohorts has been a major limitation of GWAS in other populations, which have traditionally been more reluctant to participate in genetic research and are less likely to be evaluated by memory disorder diagnosis experts. To alleviate this problem, an extended definition of cases has been utilized to increase the sample size. For instance, some studies have considered both patients with AD and mild cognitive impairment (MCI) (Driscoll *et al.* 2014, Hong *et al.* 2020) as cases. Other studies have included data from the UK Biobank and used an AD “proxy” phenotype based on parental AD status and other convenient sample cohorts in which AD status is determined through self-reporting. Two studies in which proxy AD cases accounted for more than one-half of those considered to have AD identified genome-wide significant (GWS) association with several loci that were not previously reported (Wightman *et al.* 2021, Bellenguez *et al.* 2022).

Even though utilizing the extended definition of disease status increases the statistical power, several problems still exist. First, most patients with cognitive impairment are in the MCI stage, of whom only 10%–15% progress to dementia annually (Plassman *et al.* 2008). Second, patients with MCI or potential patients with AD assigned as cases are substantially more etiologically heterogeneous than clinic-based AD cases (Escott-Price and Hardy 2022). The power loss problem because of subject misclassification or genetic heterogeneity can be alleviated by using statistical classification methods. For instance, MCI is regarded as an intermediate stage between being cognitively normal (CN) and having AD, and some patients will eventually progress to AD. An AD prediction model with good performance would allow patients with MCI to be incorporated into GWAS on AD. Several approaches for imputing phenotypes have been proposed. Hormozdiari *et al.* (2016) suggested imputing phenotypes based on a multivariate normal assumption. In addition, a deep-learning-based prediction model has been proposed to impute phenotypes using fundus images (Alipanahi *et al.* 2021). They confirmed that imputing phenotypes could increase the statistical power of GWAS. However, both studies did not explicitly consider the imputation accuracy of the phenotypes in GWAS, which differentiates our method. In particular, when imputing inter-phenotypes, the accuracy of the imputed phenotypes differs by subject, and such differences must be considered in GWAS.

Here, we present a new statistical method that incorporates imputation accuracy for MCI and phenotype-unknown subjects. We conducted a GWAS for AD involving individuals with clinically diagnosed MCI and unknown AD status. Our method improves the statistical power of GWAS for AD, and we discovered a novel significant association with rs143625563 in *LMX1A*.

## 2 Materials and methods

### 2.1 Method overview

In this study, we assumed that the disease status of subjects with MCI is unknown. To impute the disease status of subjects with missing phenotypes, we developed an AD prediction model using the AD and CN groups. Subsequently, we classified the subjects with missing phenotypes into either the AD or CN group utilizing the developed model. During this process, we calculated weights considering the accuracy of the imputed phenotypes for subjects with missing phenotypes. Finally, we conducted a case–control GWAS, which included the group with missing phenotypes; weighting imputed phenotypes (WIP) method using weighted generalized linear equation (wGEE).

To demonstrate the superiority of our method, we considered three statistical models: ordinary logistic regression (LR), GEE, and wGEE. LR is a type of a generalized linear model (GLM) and commonly used in GWASs to analyze the association between genetic variants and disease outcomes. GEE is an extension of GLM that allows modeling both continuous and categorical outcomes, and it estimates the population-averaged effects while accounting for within-group correlations. However, assuming that response is binary and the subjects are independent, GEE is equivalent to LR. wGEE assigns weights to subjects based on their importance in regression analysis and can provide an unbiased estimate when the weights are appropriately assigned. The weights are usually calculated by estimating the inverse probability of a subject dropping out at the observed time for longitudinal data (Fitzmaurice *et al.* 1995, Preisser *et al.* 2002). Here, we derived the weights based on the accuracy of the imputed phenotypes.

### 2.2 Notations and disease model

We developed a method based on a dichotomous disease phenotype; however, it can be extended to polytomous or continuous phenotypes. We assumed that there were *n_a_* cases and *n_c_* controls and that the disease status for *n_m_* subjects is missing. The disease status of subject *i* is denoted by *y_i_*, which was coded as 1 and 0 for cases and controls, respectively. For simplicity, we assumed that subjects were ordered by their disease status: the first *n_a_* subjects (*i *=* *1, …, *n_a_*) are affected, the following *n_c_* subjects (*i* = *n_a_* + 1, …, *n_a_* + *n_c_*) are unaffected, and the last *n_m_* subjects (*i* = *n_a_* + *n_c_* + 1, …, *n_a_* + *n_c_ + n_m_*) have missing phenotypes. The sets of subject indices are denoted by $N_{a}$, $N_{c}$ and $N_{m}$, respectively, and the total set of subject indices is represented by $N_{T}$ ($N_{T\;}$=$\left\{N_{a}\cup N_{c}\cup N_{m}\right\}$). The probability of subject *i* being affected is denoted as $p_{i}$, where $p_{i}$ was 1 and 0 for the cases and controls, respectively. If the disease status was unknown, $p_{i}$ was estimated. $y_{i}$ for those subjects was coded as:


yi= 1, if pi≥0.50, otherwise .

Based on these definitions, we considered LR with case/controls for the ordinary disease model, and the model is defined as follows: 


$$\mathrm{logit}\left(\mu_{i}\right)=Z_{i}^{T}\alpha +G_{i}^{T}\beta ,\;i\in \left\{N_{a}\cup N_{c}\right\};$$

where, $\mu_{i}=E\left(y_{i}|Z_{i},G_{i}\right)$, $\alpha =\left[\alpha_{0},\alpha_{1},\ldots ,\alpha_{q}\right]$ is a vector of the regression coefficients of the covariates $Z_{i}$ including an intercept, and β is a vector of the regression coefficients for SNP $G_{i}$.

### 2.3 Statistical methods

Suppose $w_{i}$ is the weight for $y_{i}$, $w_{i}$ was calculated by $p_{i}$ and is coded as:


wi= pi, if pi≥0.51-pi, otherwise , i∈ NT.

$w_{i}$ is 1 for the cases and controls; whereas, it ranges from 0.5 to 1 for subjects with missing phenotypes.

Then, we fitted a wGEE to calculate the quasi-score for the parameter $\theta ={\left(\alpha^{T},\beta^{T}\right)}^{T}$ by incorporating both cases/controls and subjects with missing phenotypes as follows:


$$U\left(\theta \right)=\left(\begin{array}{c} U_{\alpha }\left(\theta \right)\\ U_{\beta }\left(\theta \right) \end{array}\right)=\left(\begin{array}{c} \sum_{i=1}^{n_{a}+n_{c}+n_{m}}\frac{\partial \mu_{i}^{T}}{\partial \alpha }V_{i}^{-1}w_{i}\left(y_{i}-\mu_{i}\left(\theta \right)\right)\\ \sum_{i=1}^{n_{a}+n_{c}+n_{m}}\frac{\partial \mu_{i}^{T}}{\partial \beta }V_{i}^{-1}w_{i}\left(y_{i}-\mu_{i}\left(\theta \right)\right) \end{array}\right),\;i\in \;N_{T};$$

where, $V_{i}=A_{i}^{1/2}R\left(\eta \right)A_{i}^{1/2}$ and $R\left(\eta \right)$ are the working covariance matrix and working correlation matrix, respectively. In this study, an identity matrix was utilized for $R\left(\eta \right)$. Maximum likelihood estimates for α and β were calculated by solving $U\left(\hat{\theta }\right)=U\left(\hat{\alpha },\hat{\beta }\right)=0$, using Wald statistics for both parameters.

Alternatively, we used the score statistics, considering α as a nuisance parameter, and if


$$\hat{\alpha_{0}}:\;{\left.U_{\alpha }\left(\theta \right)\right|}_{\alpha =\hat{\alpha_{0}},\beta =0}=0,$$

the quasi-score of β was defined by 


$$U_{P}\left(\beta \right)=U\left(\hat{\alpha_{0}},\beta \right)=\sum_{i=1}^{n_{a}+n_{c}}\frac{\partial \mu_{i}^{T}}{\partial \beta }V_{i}^{-1}\left(y_{i}-\mu_{i}\left(\hat{\alpha_{0}},\beta \right)\right)+\sum_{i=n_{a}+n_{c}+1}^{n_{a}+n_{c}+n_{m}}\frac{\partial \mu_{i}^{T}}{\partial \beta }V_{i}^{-1}w_{i}\left(\hat{y_{i}}-\mu_{i}\left(\hat{\alpha_{0}},\beta \right)\right).$$

Then, generalized score test statistic T for testing $H_{0}:\;\beta =0$ was defined by: 


$$T={U_{P}\left(\beta =0\right)}^{T}{\left[\hat{\mathrm{var}(}U_{P}\left(\beta =0\right))\right]}^{-1}U_{P}\left(\beta =0\right).$$

### 2.4 Estimating the prediction model and affection probability

To estimate the probability of disease status, the following two-step approach was applied. First, prediction models were built with the predictors ($X_{i}$) of subjects whose disease status was known using LR, support vector machines (SVMs), random forest (RF), and gradient boosting (GB). However, the model can be extended to other machine learning or deep learning algorithms. Predictors can encompass any SNP-disease mediated variables, but SNPs should not be directly included as predictors to prevent potential false-positive issues that may arise from reusing the genetic data during association testing. Next, we selected the model with the highest area under the curve (AUC) with *k*-fold cross-validation (CV), and the probability of being affected was calculated. For instance, in LR, if the prediction model was: 


$$\mathrm{logit}\left(p_{i}\right)=X_{i}\gamma ,\;i\in N_{a}\cup N_{c},$$

and we let the estimated parameter be $\hat{\gamma }$, then the prediction model was applied to subjects whose disease status was unknown, and the estimated probability was derived as follows: 


$$p_{i}=\;{\mathrm{logit}}^{-1}\left(X_{i}\hat{\gamma }\right),\;i\in N_{m}.$$

### 2.5 Estimating bias of SNP effects due to misclassification

**Figure 1.**
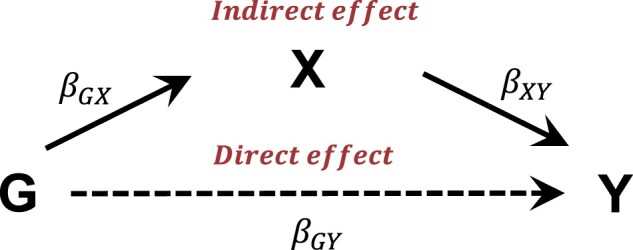
Path diagram for mediation model.

The prediction model described above should accurately predict the disease status of subjects with unknown disease status. However, the incorporation of subjects with unknown disease status into statistical analyses can induce a bias in the coefficient estimates, which may be affected by the accuracy of our prediction model. To quantify the amount of bias, we assumed that the main risk factor, G, affected the disease status, Y, of subjects with unknown disease status, predicted with the variable X. This relationship indicates G can affect Y by mediating X indirectly or directly mediating *X* through an alternative pathway (Fig. 1). In this context, predictors can be considered as mediators, and the effects of SNPs on the disease can be categorized into direct and indirect effects. We denote the regression coefficient between G and Y as $\beta_{GY}$, the SNP effects from G to X as $\beta_{GX}$, and the effects of mediator X on Y as $\beta_{XY}$. If X is continuous and there is no interaction between X and G, the total SNP effect β between G and Y becomes (Rijnhart *et al.* 2021) $\beta =\beta_{GY}+\beta_{GX}\beta_{XY}.$

Figure 1 provides a general summary of the relationship between G, X, and Y. Here, we considered the LR of Y on G, and X was not considered as a covariate. Y for subjects with unknown disease status was predicted using X, and was included in the LR. LR coefficients with and without subjects with unknown disease status are denoted by $\beta^{*}$ and β, respectively. Then, $\beta^{*}$ and β can be considered biased and true regression estimates of G for Y, respectively. The prediction model built using X without G cannot account for the direct effect ($\beta_{GY})$ of G, and its accuracy is proportional to the relative proportion of the indirect effect. If we denote the relative proportion of the direct effect by: 


$$d^{*}=\frac{\beta_{GY}}{\beta_{GY}+\;\beta_{GX}\beta_{XY}},$$

$\beta^{*}-\beta$ is expected to be proportional to $d^{*}$. We let $\gamma_{0}$ and $\gamma_{1}$ be the probabilities that true controls and cases are misclassified. $\gamma_{0}$ and $\gamma_{1}$ can be parameterized with the accuracy of the prediction model (see Supplementary Text S1). Based on these definitions, $\beta^{*}$ was approximately quantified with a scale factor (Neuhaus 1999), as follows: 


$$\beta^{*}\approx \left(\hat{B}d^{*}+\left(1-d^{*}\right)\right)\beta .$$

Here, $\hat{B}$ was obtained by:


$$\hat{B}=\frac{\left(1-\gamma_{1}-\gamma_{0}\right)}{\left(1+\gamma_{1}-\gamma_{0}\right)\left(1-\gamma_{1}+\gamma_{0}\right)}.$$

Based on this relationship, the true regression coefficient was obtained by:


$$\hat{\beta }\approx \frac{{\hat{\beta }}^{*}}{\hat{B}d^{*}+\left(1-d^{*}\right)}.$$

### 2.6 Simulation studies

We generated 10 000 replicates, and empirical type-1 error and powers were estimated with these replicates. For each replicate, we generated phenotypes and genotypes for 50 000 participants. Subsequently, 5000 cases and 5000 controls were randomly selected. Among the remaining subjects, $n_{m}/2$ cases and $n_{m}/2$ controls were selected from the case/control group, respectively, and their disease status was assumed to be unknown. We denote the probability of a person being affected by${\;p}_{i}$. The disease status $y_{i}$ of subject *i* was determined as follows: 


$$y_{i}∼\mathrm{Bernoulli}\left(p_{i}\right),\;i\;=\;1,\;\ldots ,\;50\;000.$$

If we denote SNP, sex, and 55 MRI traits of subject *i* by $G_{i}$, $D_{i}$, and $X_{i1}$, …, $X_{i55}$, respectively, then $p_{i}$ can be calculated as follows: 


$${\;p}_{i}={\mathrm{logit}}^{-1}\left\{D_{i}\alpha +\sum_{j=1}^{55}X_{ij}\gamma_{j}\;+\left(1-\nu \right)G_{i}\beta_{D}+\;\epsilon_{i}\right\}.$$

Here, we assumed that $G_{i}∼B\left(2,\;\mathrm{MAF}=0.1\right)$. For α and $\gamma_{j}$, we utilized LR estimates from our real-data analyses. $D_{i}$ was randomly assigned to either male or female subjects. We assumed that 55 MRI traits were available, and ${X\prime }_{ij}$, for *j*-th region of interest (ROI) was generated from the normal distributions. The first *k* MRI traits were assumed to be affected by the SNP as follows: 


$$X_{ij}=\left\{\begin{array}{cc} {X'}_{ij}-\;\nu \;\beta_{I}G_{i}, & j=1,\ldots ,k\\ {X'}_{ij}, & j=k+1,\ldots ,55 \end{array}\right.$$

where


$${X^{'}}_{ij}\;∼\;N\left(0,1\right),\;j=1,\ldots ,55.$$

This simulation setting indicates that SNPs can affect the disease status directly and indirectly through the first k MRI traits. ν was utilized to parameterize the weight of the indirect effect of SNP on disease status, and was set to 0, 0.3, 0.5, 0.8, and 1. We considered k=10,30. $\epsilon_{i}$ indicates the unobserved environment effect and was generated from $N\left(0,1\right)$. $\beta_{D}$ and $\beta_{I}$ indicate the parameters for direct and indirect effects, respectively, and they were obtained using the following two different measures $h_{d}^{2}$ and $h_{i}^{2}$, respectively: 


$$\begin{array}{c} h_{d}^{2}=\frac{\beta_{D}^{2}var(G)}{\alpha^{2}var\left(D\right)+\;\sum_{j=1}^{55}\gamma_{j}^{2}var\left(X_{j}\right)+\;\beta_{D}^{2}var\left(G\right)+\mathrm{var}(\epsilon )},\\ h_{i}^{2}=\frac{\;\sum_{j=1}^{55}\gamma_{j}^{2}\beta_{I}^{2}var(G)}{\alpha^{2}var\left(D\right)+\;\sum_{j=1}^{55}\gamma_{j}^{2}var\left(X_{j}\right)\;+\;\sum_{j=1}^{55}\gamma_{j}^{2}\beta_{I}^{2}var(G)+\mathrm{var}(\epsilon )}. \end{array}$$

$h_{d}^{2}\;\mathrm{and}\;h_{i}^{2}$ were set to (0,0) and (0.0001,0.001), respectively. The results from the former and latter were used to estimate empirical type-1 error and empirical power, respectively. Accordingly, $\left(\beta_{D},\;\beta_{I}\right)$ became (0,0) and (0.049,0.088), respectively.

All analyses were conducted using the Statmodels (v.0.13.2) (Seabold and Perktold 2010) and Scikit-learn (v.1.1.1) (Pedregosa *et al.* 2011) libraries in Python (v.3.8).

### 2.7 Subject classification and characteristics

A total of 5193 participants were recruited by the Gwangju Alzheimer’s and Related Dementia (GARD) study at Chosun University in Gwangju, South Korea. The clinical diagnosis of AD was made according to the National Institute of Neurological and Communicative Disorders and Stroke-Alzheimer Disease and Research Disorders Association (NINCDS-ADRDA) criteria (Park *et al.*, 2019). The sample consisted of 1241 patients with AD, 1256 with MCI, 2382 CN subjects, and 314 subjects who were not classified because of missing information (i.e. unknown) (Table 1). All study volunteers or authorized guardians of cognitively impaired individuals provided written informed consent before participation.

**Table 1.** Descriptive statistics. For GWAS, subjects consist of 1241 AD subjects, 2382 CN subjects, and 1570 Other subjects. The 1570 Other subjects consist of 1256 MCI subjects and 314 unknown subjects. “Unknown” indicates subjects with missing diagnosis. MMSE, Mini-Mental Status Examination.

Group	Total	CN	AD	Other		
				Total	MCI	Unknown
*N*	5193	2382	1241	1570	1256	314
Age, mean (SD)	73.4 (6.8)	72.7 (6.7)	74.8 (7.1)	73.5 (6.8)	73.2 (6.8)	74.7 (6.3)
Male sex, *n* (%)	2101 (40.4)	928 (39.0)	479 (38.6)	694 (44.2)	573 (45.6)	121 (38.5)
Education, mean (SD)	9.7 (4.8)	9.8 (4.7)	7.7 (5.0)	9.8 (4.9)	10 (4.8)	9.2 (5.0)
MMSE mean, (SD)	25.8 (3.9)	27.2 (2.3)	18.6 (5.8)	25.6 (3.2)	25.7 (3.1)	25.5 (4.2)
Apoe e4 carrier, *n* (%)	1582 (30.4)	624 (26.2)	545 (43.9)	413 (26.3)	341 (27.1)	72 (22.9)

### 2.8 Statistical methods for AD prediction model with GARD cohort and evaluation

The AD prediction models were built with information from 369 subjects with AD and 2267 CN subjects whose characteristics are shown in Supplementary Text S2 and Supplementary Table S1. These models included 55 MRI traits, including 31 cortical thickness-related traits and 24 subcortical volume measures for brain ROIs, and five SNSB cognitive test scores (one test each for measures of attention, language, visuospatial function, memory, and frontal/executive function), log-transformed intracranial volume, and sex. The prediction model was generated with four different algorithms including penalized LRs (penalized LRs), GB, RFs, and SVM.

To evaluate the performance of different models, 5-fold nested CV was applied to calculate the AUCs. Outer CV was used to estimate the test AUCs, and inner CV was used to optimize the hyperparameters for our prediction model. Details of optimizing hyperparameters are shown in Supplementary Text S3. Finally, the model with the best AUC was fitted to all CN subjects and subjects with AD. The overall scheme for building and evaluating the models is shown in Supplementary Fig. S1. All analyses were conducted using the Scikit-learn (v.1.1.1) library in Python (v.3.8).

### 2.9 Genome-wide association studies with GARD cohort

A GWAS for AD was conducted using three different models. Ordinary LR (LR) models were applied to a sample including only AD cases and CN controls. IP and WIP method were employed to test association for AD in enlarged samples that also included MCI and AD status unknown subjects using GEE and wGEE. In these analyses, the weights for AD cases and CN subjects were 1, whereas the estimated AD probabilities were used for MCI and status unknown subjects. The detailed procedure for the overall scheme is summarized in Supplementary Fig. S2 (see details for genotyping, quality control, and imputation procedures in Supplementary Text S4). Age, sex, and the first three PCs were included as covariates in all models. The GWS level was set at 5×$10^{-8}$. LR and IP analyses were performed using PLINK (v.1.90 beta) (Purcell *et al.* 2007), and WIP method were conducted in Python (v.3.8) using the Statmodel library (v.0.13.2). We used LocusZoom (Pruim *et al.* 2010) to generate regional plots and R software (v.3.7) (R Development Core Team, Vienna, Austria) to generate QQ and Manhattan plots.

To estimate the bias introduced by the misclassification probabilities and correct the odds ratios (ORs) for SNPs in the GWAS, the true ratio of cases among MCI/status unknown subjects (ρ) was assumed to be 0.15 based on the conversion rate from MCI to dementia per year (Petersen *et al.* 2001). Furthermore, the prediction performance for MCI/status unknown subjects ($\pi_{1},\pi_{0}$) was adjusted using the sensitivities and specificities of the simulation results to calculate the disease status misclassification probabilities. The proportion of direct effects ($d^{*}$) was estimated by dividing the SNP heritability adjusted for MRI traits by the SNP heritability without adjustment.

## 3 Results

### 3.1 Type-1 error assessment and statistical power comparison with simulated data

We compared the empirical type-1 errors and power estimates at the nominal significance level α, for α=0.05, 0.01, and 0.001. Type-1 errors and powers were defined as the ratio of subjects whose *P*-values were smaller than the thresholds. As shown in Table 2, we assumed that $\left(\beta_{D},\;\beta_{I}\right)$=(0, 0), and type-1 errors for all methods did not result in inflation at the nominal level. Moreover, the simulation results with SNP effect sizes $\left(\beta_{D},\;\beta_{I}\right)$=(0, 0) were not affected by various $n_{m}$ and ν, which indicated that our simulation settings were valid. For power comparison, we assumed $h_{i}^{2}=0.001,\;h_{d}^{2}=0.0001$, and in this case, the SNP effect sizes became $\left(\beta_{D},\;\beta_{I}\right)$=(0.049, 0.081).

**Table 2.** Estimated type-1 error rates and powers with simulation data. (A) Estimated type-1 error rates by logistic regression (LR) with only case/control group [n=10 000 ($n_{c}=5000$, $n_{a}=5000$)], imputed phenotypes (IP) with GEE, and weighting imputed phenotypes (WIP) with wGEE including missing group [$n=11\;000,\;12\;500,\;15\;000\;(n_{c}=5000$, $n_{a}=5000$, $n_{m}=1000,\;2500,\;5000$)] were estimated at the nominal significance level α. K indicates the number of MRI traits affected by the SNP.

	LR with only case/control group			IP including missing group			WIP including missing group		
$n_{m}(n_{m}/n)$	0.05	0.01	0.001	0.05	0.01	0.001	0.05	0.01	0.001
1000 (0.09)	0.0507 (0.0022)	0.0104 (0.0011)	0.0015 (0.0003)	0.0493 (0.0023)	0.0105 (0.0012)	0.0014 (0.0003)	0.0481 (0.0024)	0.0105 (0.0011)	0.0011 (0.0003)
2500 (0.20)				0.0485 (0.0021)	0.0105 (0.0010)	0.0017 (0.0002)	0.0511 (0.0023)	0.0101 (0.0011)	0.0018 (0.0002)
5000 (0.33)				0.0498 (0.0020)	0.0092 (0.0011)	0.0015 (0.0004)	0.0505 (0.0021)	0.0094 (0.0011)	0.0013 (0.0003)

Table 3 shows that the power estimates of all methods increased proportionally with ν for a fixed k (k=10). The indirect effect $\beta_{I}$ was set to be larger than the direct effect $\beta_{D}$, and when ν increased, the indirect effect of the SNPs on the disease increased. Assuming that all SNPs directly affected the disease (ν=0), the logistic method was statistically more powerful than the IP and WIP methods including subjects with missing AD status. Furthermore, when additional $n_{m}$ subjects with missing AD status were utilized, the power of the IP and WIP methods worsened. However, if ν increases, then the IP and WIP methods using subjects with unknown AD status outperform the logistic method. When ν=0.3, the power of the IP and WIP methods was comparable to that of the logistic method, and if ν≥0.5, then the IP and WIP methods performed better than the logistic model. Concerning the change in $n_{m}$, for a fixed ν (ν= 0.5, 0.8 1), the IP and WIP methods were more powerful as the number of $n_{m}$ increased, and these positive effects due to the sample size become larger as ν increased. In addition, the WIP method had slightly higher power than the IP model, without considering the weights for all values of ν and $n_{m}$.

**Table 3.** Estimated type-1 error rates and powers with simulation data. (B) Estimated powers by logistic regression (LR) with only case/control group [n=10 000 ($n_{c}=5000$, $n_{a}=5000$)], imputed phenotypes (IP) with GEE, and weighting imputed phenotypes (WIP) with wGEE including missing group [$n=11\;000,\;12\;500,\;15\;000\;(n_{c}=5000$, $n_{a}=5000$, $n_{m}=1000,\;2500,\;5000$)] were estimated at the nominal significance level α. K indicates the number of MRI traits affected by the SNP.

	***K*** **=** **10**	LR with only case/control group			IP including missing group			WIP including missing group		
*v*	$n_{m}(n_{m}/n)$	0.05	0.01	0.001	0.05	0.01	0.001	0.05	0.01	0.001
0	1000 (0.09)	0.1590(0.0036)	0.0520(0.0023)	0.0092(0.0009)	0.1494(0.0036)	0.0463(0.0019)	0.0086(0.0009)	0.1496(0.0037)	0.0469(0.0020)	0.0086(0.0009)
	2500 (0.20)				0.1385(0.0030)	0.0417(0.0019)	0.0068(0.0008)	0.1438(0.0031)	0.0445(0.0021)	0.0071(0.0008)
	5000 (0.33)				0.1203(0.0033)	0.0350(0.0016)	0.0059(0.0008)	0.1275(0.0032)	0.0390(0.0017)	0.0064(0.0009)
0.3	1000 (0.09)	0.1820(0.0041)	0.0658(0.0025)	0.0112(0.0011)	0.1806(0.0042)	0.0648(0.0025)	0.0123(0.0011)	0.1830(0.0042)	0.0653(0.0026)	0.0132(0.0012)
	2500 (0.20)				0.1762(0.0038)	0.0615(0.0026)	0.0123(0.0013)	0.1817(0.0040)	0.0641(0.0026)	0.0128(0.0014)
	5000 (0.33)				0.1696(0.0037)	0.0573(0.0026)	0.0130(0.0011)	0.1817(0.0040)	0.0616(0.0027)	0.0141(0.0012)
0.5	1000 (0.09)	0.2099(0.0038)	0.0718(0.0024)	0.0147(0.0012)	0.2086(0.0037)	0.0720(0.0024)	0.0144(0.0011)	0.2123(0.0037)	0.0739(0.0026)	0.0150(0.0012)
	2500 (0.20)				0.2104(0.0038)	0.0765(0.0027)	0.0158(0.0015)	0.2163(0.0038)	0.0803(0.0026)	0.0160(0.0015)
	5000 (0.33)				0.2181(0.0037)	0.0799(0.0027)	0.0165(0.0016)	0.2259(0.0037)	0.0839(0.0028)	0.0185(0.0016)
0.8	1000 (0.09)	0.2346(0.0042)	0.0898(0.0029)	0.0205(0.0013)	0.2444(0.0042)	0.0946(0.0029)	0.0221(0.0013)	0.2492(0.0041)	0.0970(0.0030)	0.0214(0.0013)
	2500 (0.20)				0.2628(0.0041)	0.1066(0.0030)	0.0258(0.0015)	0.2698(0.0040)	0.1104(0.0032)	0.0269(0.0016)
	5000 (0.33)				0.2891(0.0045)	0.1184(0.0032)	0.0299(0.0017)	0.3028(0.0045)	0.1266(0.0032)	0.0327(0.0016)
1.0	1000 (0.09)	0.2546(0.0047)	0.1063(0.0035)	0.0228(0.0014)	0.2737(0.0043)	0.1140(0.0033)	0.0253(0.0016)	0.2760(0.0042)	0.1180(0.0034)	0.0253(0.0016)
	2500 (0.20)				0.3061(0.0043)	0.1336(0.0036)	0.0326(0.0019)	0.3153(0.0044)	0.1376(0.0035)	0.0339(0.0020)
	5000 (0.33)				0.3503(0.0047)	0.1571(0.0039)	0.0446(0.0022)	0.3667(0.0048)	0.1682(0.0040)	0.0505(0.0023)

The results for k=30 are listed in Supplementary Table S2. The results for k=30 were consistent with those for k=10, but the IP and WIP methods tended to have better power than the LR as long as ν≥0.3.

### 3.2 Estimation of SNP coefficients and bias correction with simulated data

SNP regression coefficients were estimated using ordinary LR, IP, and WIP: ${\hat{\beta }}_{LR}$, ${\hat{\beta }}_{IP}$, and ${\hat{\beta }}_{\mathrm{WIP}}$, respectively, and compared across methods for effect sizes $\left(\beta_{D},\;\beta_{I}\right)$=(0.049, 0.081) (Supplementary Table S3). The coefficients for all methods tended to increase proportionally with ν for the same reasons as power. The coefficients for the IP and WIP methods (${\hat{\beta }}_{IP}$, ${\hat{\beta }}_{\mathrm{WIP}})$ had a downward bias compared to those of the logistic method. The underestimation of SNP coefficients worsened with lower ν and higher $n_{m}$ values. However, for ν= 1 (no direct effects), ${\hat{\beta }}_{IP}$ and ${\hat{\beta }}_{\mathrm{WIP}}$ were approximately the same as ${\hat{\beta }}_{LR}$, regardless of the sample size ($n_{m}$).

We also calculated the bias estimate ($\hat{B}$) to correct the coefficient estimates for the IP and WIP methods. For each simulation, we obtained the misclassification probabilities for the subjects with missing diagnosis status using the prediction errors and true simulation values, and the proportion of direct effects with various K and ν. Finally, we derived $\hat{B}$ and the adjusted $\hat{B}$ as ${\hat{\beta }}_{IP}$ and ${\hat{\beta }}_{\mathrm{WIP}}$, respectively, to obtain the bias-adjusted estimations (${\hat{\beta }}_{IP}^{\mathrm{adj}}$, ${\hat{\beta }}_{\mathrm{WIP}}^{\mathrm{adj}}$). After bias correction, the adjusted estimates for the IP and WIP methods were larger than the nonadjusted estimates in all situations and close to ${\hat{\beta }}_{LR}$. However, for ν≤0.3, adjusted estimates showed a downward bias when the proportion of subjects with missing diagnosis status increased. Supplementary Table S4 shows the results of SNP estimates and bias correction for k=30.

### 3.3 Application of the AD prediction model to the GARD cohort

**Figure 2.**
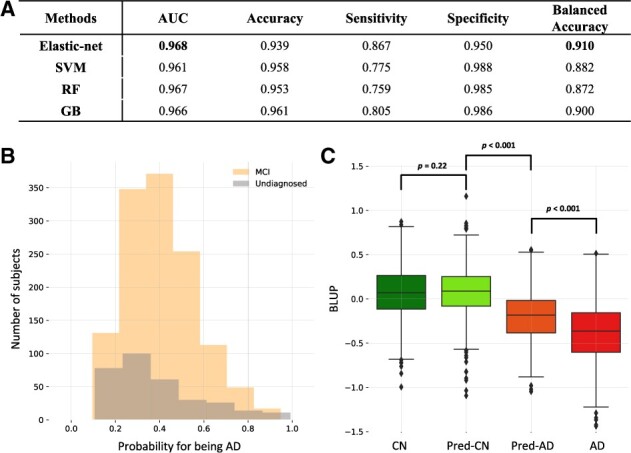
Validation of AD prediction model for GARD cohort. (A) Prediction results of different machine-learning methods; RF, random forest; GB, gradient boosting; SVM, support vector machine. (B) The histograms compare the distributions of probabilities of being AD for MCI/unknown subjects. (C) The boxplots of BLUPs for four groups; CN subjects, MCI/unknown subjects who were predicted as CN subjects (Pred-CN), MCI/unknown subjects who were predicted as AD subjects (Pred-AD), and AD subjects.

Figure 2A shows the prediction results obtained from models including cognitive and MRI measures among the CN and patients with AD. The results showed that the penalized LR had the best AUC and balanced accuracy (AUC = 0.968, balanced accuracy = 0.910). The prediction model built with penalized LR was applied to 1570 MCI and AD status unknown subjects, among whom 388 MCI/status unknown subjects were classified under AD (MCI 311, unknown 77) and 1182 subjects were classified under CN (MCI 945, unknown 237). The distributions of the probability of AD in status unknown subjects are shown in Fig. 2B. The MCI and status unknown groups were on an average more similar to the CN group with mean AD probabilities of 0.41 ± 0.16 and 0.39 ±0.20, respectively.

### 3.4 SNP AD heritability accounted for by MRI and cognitive traits

The AD heritability accounted for by SNPs was estimated to be 67% (*P* < .001) after adjusting for sex, age, and PCs. However, heritability decreased to 36% when MRI traits and cognitive test scores were included in the model (*P* < .001). These results indicate that MRI traits and cognitive score tests are associated with a substantially large number of disease susceptibility loci (Methods are in Supplementary Text S5).

### 3.5 Evaluating prediction results with genetics

To evaluate the performance of the AD prediction model applied to MCI and status unknown subjects, CN subjects, AD cases, and MCI/status unknown subjects who were predicted as CN (Pred-CN) and AD subjects (Pred-AD) were compared using the best linear unbiased prediction estimations (BLUPs) for hippocampal volume. Details of calculating BLUPs are in Supplementary Text S6). Figure 2C shows that the mean difference of BLUPs between Pred-CN and Pred-AD subjects was significantly different (*P*<.001). Furthermore, the distributions of BLUPs for Pred-CN and CN subjects were not significantly different (*P* = .22). The mean of BLUPs was higher for Pred-AD subjects than for AD cases (*P* <.001). These results suggest that the classification of MCI and status unknown subjects is reliable.

### 3.6 Contribution of MCI and subjects with unknown AD status to GWAS findings

We conducted three separate GWAS for AD. One GWAS included 1241 patients with AD and 2382 CN subjects. The other two GWAS included the MCI and status unknown subjects who were classified, yielding a total of 1629 AD cases and 3564 CN subjects (Table 4 and Supplementary Figs S3 and S4). In the analysis including the clinically diagnosed AD cases and controls only, we identified the GWS association of AD with the *APOE* SNP encoding ε4 (rs429358, *P*=$9.4\times 10^{-41}$). The significance of this finding increased in the analyses including the MCI/status unknown subjects classified under AD or CN using the IP (*P*=$4.0\times 10^{-45}$) and WIP (*P*=$7.4\times 10^{-49}$) methods. Similar patterns of GWS association were observed for SNPs in other genes in the *APOE* region. In addition, we identified a novel association with rs143625563 located in *LMX1A* (LIM homeobox transcription factor 1 alpha) in the analyses including the additional subjects who were classified using the IP (*P*=$1.4\times 10^{-8}$) and WIP (*P*=$5.3\times 10^{-8}$) methods, respectively (Supplementary Fig. S5A). This association was several orders of magnitude less significant in the analyses without the newly classified subjects (*P*=$6.9\times 10^{-6}$). Notably, rs3829687 located in the previously established AD gene *ABCA7* showed mild association upon analysis of the clinically diagnosed AD cases and controls (*P*=$6.4\times 10^{-6}$) and in the analysis with the newly classified subjects (*P*=$3.5\times 10^{-7}$) using the IP method but was marginal (*P*=$8.9\times 10^{-8}$) using the WIP method (Supplementary Fig. S5B).

**Table 4.** Significant AD GWAS results (*P*<1.0×10^−7^) for logistic regression (LR) with only CN-AD, imputed phenotypes (IP) with GEE, and weighting imputed phenotypes (WIP) with wGEE including MCI/unknown.^a^

CHR	BP	SNP	MA	IQS	LR with only CN-AD			IP including MCI/unknown				WIP including MCI/unknown				Gene
					OR	*P*	MAF	OR	${\mathrm{OR}}^{\mathrm{adj}}$	*P*	MAF	OR	${\mathrm{OR}}^{\mathrm{adj}}$	*P*	MAF	
19	45406673	rs10119	A	0.89	2.4	1.6e-40	0.18	2.2	2.3	2.4e-45	0.17	2.3	2.4	6.1e-49	0.17	*TOMM40*
19	45411941	rs429358	C	0.97	2.5	9.4e-41	0.17	2.2	2.4	4.0e-45	0.16	2.3	2.5	7.4e-49	0.16	*APOE*
19	45421254	rs12721046	A	0.85	2.3	4.4e-35	0.17	2.1	2.2	2.6e-39	0.16	2.2	2.3	3.2e-42	0.16	*APOC1*
19	45388130	rs34342646	A	G	2.2	2.8e-29	0.15	2.0	2.1	2.2e-32	0.14	2.1	2.2	1.4e-34	0.14	*NECTIN2*
1	165219294	rs143625563	G	0.83	1.5	6.9e-6	0.07	1.6	1.6	1.4e-8	0.07	1.6	1.6	5.3e-8	0.07	*LMX1A*
19	1053299	rs3829687	T	0.97	0.5	6.4e-6	0.38	0.8	0.8	3.5e-7	0.40	0.8	0.8	8.9e-8	0.40	*ABCA7*

a Chr, chromosome; BP, base pair; MA, minor allele; MAF, minor allele frequency; IQS, imputation quality score; G, genotyped SNP; OR, odds ratio; ${\mathrm{OR}}^{\mathrm{adj}}$, adjusted odds ratio.

The estimated ORs of our methods using MCI/status unknown subjects for GWS SNPs were underestimated compared to those of LR (Table 3). We calculated the adjusted ORs (${\mathrm{OR}}^{\mathrm{adj}}$) for the IP and WIP methods by estimating bias with several assumptions, and the estimates were close to the estimates of LR.

## 4 Discussion

The sample size is a major contributor to the power of GWAS for complex diseases. We developed a method using a WIP approach that boosts power by allowing the incorporation of subjects with intermediate or missing phenotypes who are assigned a probability of disease status based on disease-related endophenotypes. We applied this method to a GWAS for AD in which subjects with MCI or an unknown AD status were assigned an AD probability based on a set of brain MRI and cognitive test parameters. Evaluation of the performance of the WIP method through simulation studies showed that it minimized type-1 errors and had superior statistical power compared to LR, which is commonly used in GWAS. The accuracy of the AD probability calculation when applied to subjects with MCI and an unknown AD status, as well as the increase in power, are exemplified by the increased significance of associations we observed for established AD loci, including several genes in the *APOE* region and *ABCA7*.

Utilization of this method for GWAS of complex diseases requires careful investigation of the endophenotypes that might be included in models for predicting disease status among those with intermediate or unknown disease status. Studies have reported moderate to high SNP heritability for brain structure (26%–88%) (Matura *et al.* 2014, Hibar *et al.* 2015, van der Lee *et al.* 2019) and cognitive function (20%–46%) (Davies *et al.* 2016), and their strong associations with AD (Raji *et al.* 2009, Suzuki *et al.* 2019). We estimated the SNP heritability of AD in the GARD study dataset to be 0.67. Reduction in the heritability estimate to 0.36 after adjusting for MRI traits and cognitive test scores suggests that the effects of SNPs on AD risk are mediated by brain structure and function to some extent. To validate our prediction model, we showed that the distribution of PRS for MCI/status unknown subjects who had a high probability of AD was significantly different from the PRS distribution for subjects who were predicted to likely be CN.

We identified GWS association with SNP rs143625563 in a novel gene, *LMX1A*, and a highly suggestive association with a variant in an established AD gene, *ABCA7*. These associations were not evidenced in a previous study using clinically diagnosed AD cases and controls in the same dataset (Kang *et al.* 2021). *LMX1A* is known to be a key transcription factor associated with dopamine (DA) neurogenesis in the midbrain (Andersson *et al.* 2006, Yan *et al.* 2011, Doucet-Beaupré *et al.* 2015, Rolstad *et al.* 2015) and is linked to neurodevelopmental and neurodegenerative DA-related diseases such as Parkinson’s disease (Cai *et al.* 2009, Laguna *et al.* 2015). Several studies have reported that DA is involved in AD pathogenesis, and DA dysfunction is associated with amyloid deposition (Perez *et al.* 2005, Himeno *et al.* 2011). Reduced DA expression is correlated with atrophy of the prefrontal cortex and hippocampus (Kumar and Patel 2007). One recent study of working memory training in amnestic and non-amnestic patients with MCI found that the non-amnestic memory group had larger gains than the amnestic MCI group, especially in *APOE* ε4 noncarriers who had the *LMX1A* rs4657412 AA genotype (Hernes *et al.* 2021). This variant is located approximately 42 kb from rs143625563 but was not highly correlated with our finding (r=0.05). AD has been associated with other *ABCA7* variants in non-Hispanic White (Jansen *et al.* 2019, Kunkle *et al.* 2019), African American (Reitz *et al.* 2013), and Chinese (Wang *et al.* 2022) populations. In addition, these variants [rs4147929 (Jansen *et al.* 2019, Wang *et al.* 2022), rs3752243 (Kunkle *et al.* 2019), rs115550680 (Reitz *et al.* 2013), rs3764650 (Wang *et al.* 2022)] are apart by approximately 10, 0.7, 3, and 7 kb from rs3829687, respectively, and correlated with our findings (r =0.34, 0.97, not in our discovery dataset and 0.25, respectively).

The proposed method for assigning the probability of disease based on endophenotypic measures has several limitations. First, although it performed well in terms of statistical power, the estimates of SNP coefficients showed a slight downward bias compared with those from the base method. This phenomenon resulted from the misclassification of subjects with an unknown disease status, and if the prediction model is not accurate, then the underestimation can be substantial. We quantified the bias in terms of prediction errors, the number of subjects with unknown disease status, and the ratio of direct effects between SNPs and diseases. Such quantification was validated through simulation studies and real-world data, and it should be noted that the result of the hypothesis testing was not affected. However, to adjust the estimates, unknown parameters that cannot be easily estimated must be specified. Second, more sophisticated methods, such as deep learning, can be utilized to improve the prediction accuracy of disease status for missing phenotypes. Some deep-learning algorithms with convolutional neural networks have been reported to perform with better than 95% accuracy using raw MRI data (Basaia *et al.* 2019, Zhang *et al.* 2021). However, compared to traditional machine-learning models, as the architecture of deep neural networks becomes deeper to improve the accuracy, the model becomes miscalibrated (Guo *et al.* 2017). Therefore, in our framework, we need to further consider how to calibrate the model because the probabilities were utilized as weights for the GWAS. Third, our findings should be replicated in independent datasets. Further experimental and clinical studies with additional data are necessary to demonstrate the effects of rs3829687 and rs143625563 on AD.

In conclusion, we showed that the statistical power of GWAS can be improved by utilizing a prediction model to quantify AD risk for subjects with MCI and an unknown AD status. Our results illustrate the practical importance of the proposed method and may improve our understanding of the genetic basis and pathogenesis of AD.

## Ethics approval and consent to participate

The study was approved by the Institutional Review Boards of Chosun University Hospital (CHOSUN 2013—12–018‐068) and Chonnam National University (CNUH‐2019‐279). Written informed consent was obtained from each participant or their legal guardian.

## Supplementary Material

btad534_Supplementary_Data

## Data Availability

The data underlying this article will be shared on reasonable request to the corresponding author.
