# Supplementary material for: Machine learning-based quantification for disease uncertainty increases the statistical power of genetic association studies
Source: Bioinformatics. 2023 Sep 4;39(9):btad534. doi: 10.1093/bioinformatics/btad534 (PMC10539075; doi:10.1093/bioinformatics/btad534)
Supplement: btad534_Supplementary_Data [file btad534_supplementary_data.pdf]

Machine learning-based quantification for disease uncertainty increases the statistical power of genetic association studies

Supplementary Text 1

We denote the true disease status of the true disease status for total, cases, controls, and unknown disease status groups by T, T^a, T^c and T^m, and the predicted diagnosis status by Y, Y^a, Y^c and Y^m, respectively. We assume that there is no subject whose disease status is incorrectly known, which means $P(Y^a = 0, T^a = 1) = 0$ and $P(Y^c = 1, T^c = 0) = 0$. We let $\rho (0 \leq \rho \leq 1)$ be the ratio of true cases among subjects with unknown disease status, and π_1, π_0 indicate the sensitivity and specificity of the prediction model as follows:

$$\pi_1 = P(Y^m = 1 \mid T^m = 1), \pi_0 = P(Y^m = 0 \mid T^m = 0).$$

Then, the misclassification probabilities of the disease status for true cases and controls can be parameterized as:

$$\gamma_1 = P(Y = 0 \mid T = 1) = \frac{P(Y = 0, T = 1)}{P(T = 1)}$$

$$= \frac{n_a P(Y^a = 0 | T^a = 1) + \rho n_m P(Y^m = 0 | T^m = 1)}{n_a + \rho n_m}$$

$$= \frac{\rho n_m}{n_a + \rho n_m}(1 - \pi_1)$$

$$\gamma_0 = P(Y = 1 \mid T = 0) = \frac{P(Y = 1, T = 0)}{P(T = 0)}$$

$$= \frac{n_c P(Y^c = 1 | T^c = 0) + (1 - \rho) n_m P(Y^m = 1 | T^m = 0)}{n_c + (1 - \rho) n_m}$$

$$= \frac{(1-\rho)n_m}{n_c + (1-\rho)n_m}(1-\pi_0).$$

Supplementary Text 2 Descriptive statistics of participants for building prediction models

We built the AD prediction models with 369 patients with AD and 2,267 CN subjects. For patients with AD, we only obtained the cognitive tests and brain MRIs for 369 patients among 1,241 patients with AD in the discovery dataset. In total, 2,267 CN subjects consists of 1,670 subjects from discovery dataset and 597 subjects from not discovery dataset but GARD cohort. Five hundred and ninety-seven subjects did not undergo blood tests for genotyping but underwent cognitive tests and brain MRIs; therefore, we only utilized them to build prediction models. Supplementary Table 3 shows the demographic information for samples to build prediction models.

Supplementary Text 3 Optimizing hyper-parameters for machine learning models

Each method has multiple hyperparameters: lambda and alpha for penalized LR; learning rate, number of estimators, and max depth for GB; number of estimators and max depth for RF; and Gamma and C for SVM. They were optimized to achieve the best prediction AUC with grid search. For instance, lambda and alpha for penalized LR varied between 0 and 100 and between 0 and 1, respectively (lambda = [0.001, 0.01, 0.02, 0.1, 0.5, 1, 10, and 100] and alpha = [0, 0.1, 0.2, 0.3, 0.4, 0.5, 0.6, 0.7, 0.8, 0.9, and 1]). For GB, we considered learning rate = [0.0001, 0.001, 0.01, 0.05, 0.1, and 0.2], number of estimators = [10, 100, 500, and 1,000], and max depth = [1, 3, 5, and 10]. For RF, we considered number of estimators = [100, 300, 500, 1,000, and 2000] and max depth = [3, 5, 10, 20, 50, 100, and 1000]. For SVM, gamma and C ranged

from 0.001 to 1 and from 0.001 to 10, respectively.

Supplementary Text 4 Genotyping, quality control, and imputation procedures

Subjects were genotyped using the Affymetrix Axim KORV 1.1 array (Affymetrix® Axiom KORV1.1, Santa Clara, CA, USA) (Moon, et al., 2019). Genotype calling was performed using the K-medoid method (Seo, et al., 2019). Genotype imputation was conducted using the Haplotype Reference Consortium v1.1 reference panel (McCarthy, et al., 2016) and the Michigan Imputation Server (Das, et al., 2016).

A total of 5,193 subjects were genotyped with an Affymetrix Axim KORV 1.1(Affymetrix®Axiom KORV1.1, Santa Clara, CA, USA) [3]. Subjects with a low genotyping call rate (<95%), and low or high genome-wide heterozygosity (±3 standard deviation from the average heterozygosity rate) were excluded. In addition, SNPs were eliminated if the call rate was <95%, Hardy−Weinberg Equilibrium (HWE) test p-value was $<1\times10^{-6}$, or minor allele frequency (MAF) was <1%. All quality control procedures were performed using PLINK. To increase the genotyping coverage, genotypes were imputed using Haplotype Reference Consortium (HRC) v1.1 reference [4] from the Michigan Imputation Server [5]. After imputation, the quality SNPs were excluded if the INFO score was <0.5, genotype call rate was <0.98, or p-value for HWE $<1\times10^{-6}$. After quality control, 6,623,671 SNPs were considered for the GWAS.

Supplementary Text 5 Estimating SNP heritability

A linear mixed model was used to calculate the SNP heritability of AD and CN subjects with GCTA (v.1.93.0. beta3) (Yang, et al., 2011). Sex, age, first three principal component (PC)

scores estimated from the genetic relationship matrix, and 55 MRI traits and five SNSB cognitive test scores were included as covariates.

Supplementary Text 6 Best linear unbiased prediction estimation

To assess the performance of the AD prediction model applied to MCI/unknown subjects, we calculated the total genetic effect for hippocampal volume using the best linear unbiased prediction (BLUP) method (Henderson, 1975) using SNP data from AD, CN, and MCI/unknown subjects who were predicted as having AD or CN. The model included terms for sex, age, and logICV and was evaluated using GCTA (v.1.93.0. beta3) (Yang, et al., 2011).

Supplementary Figure 1. Procedure for building and evaluating AD prediction models.

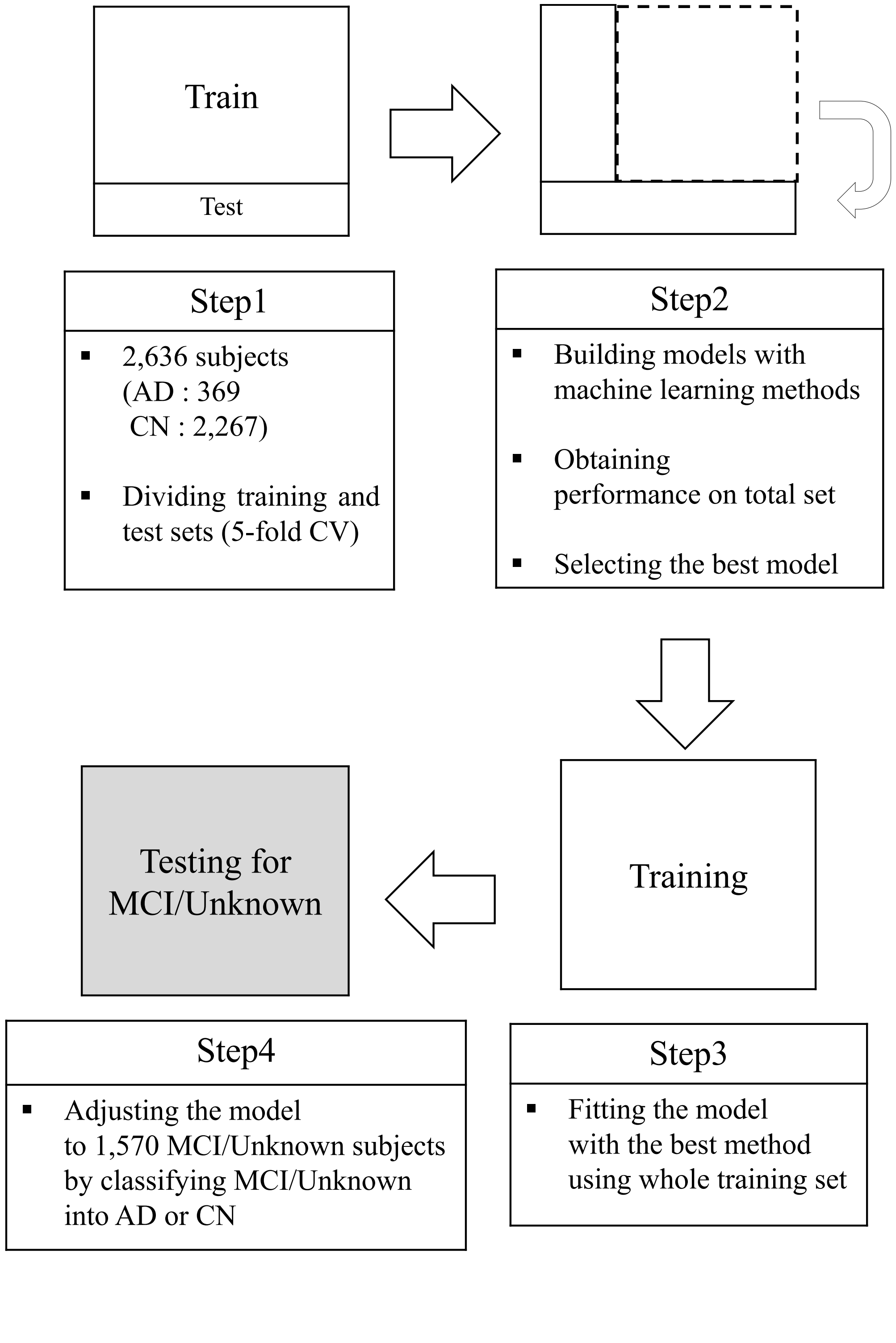

Supplementary Figure 2. Overall scheme for genome-wide association studies with GARD cohort.

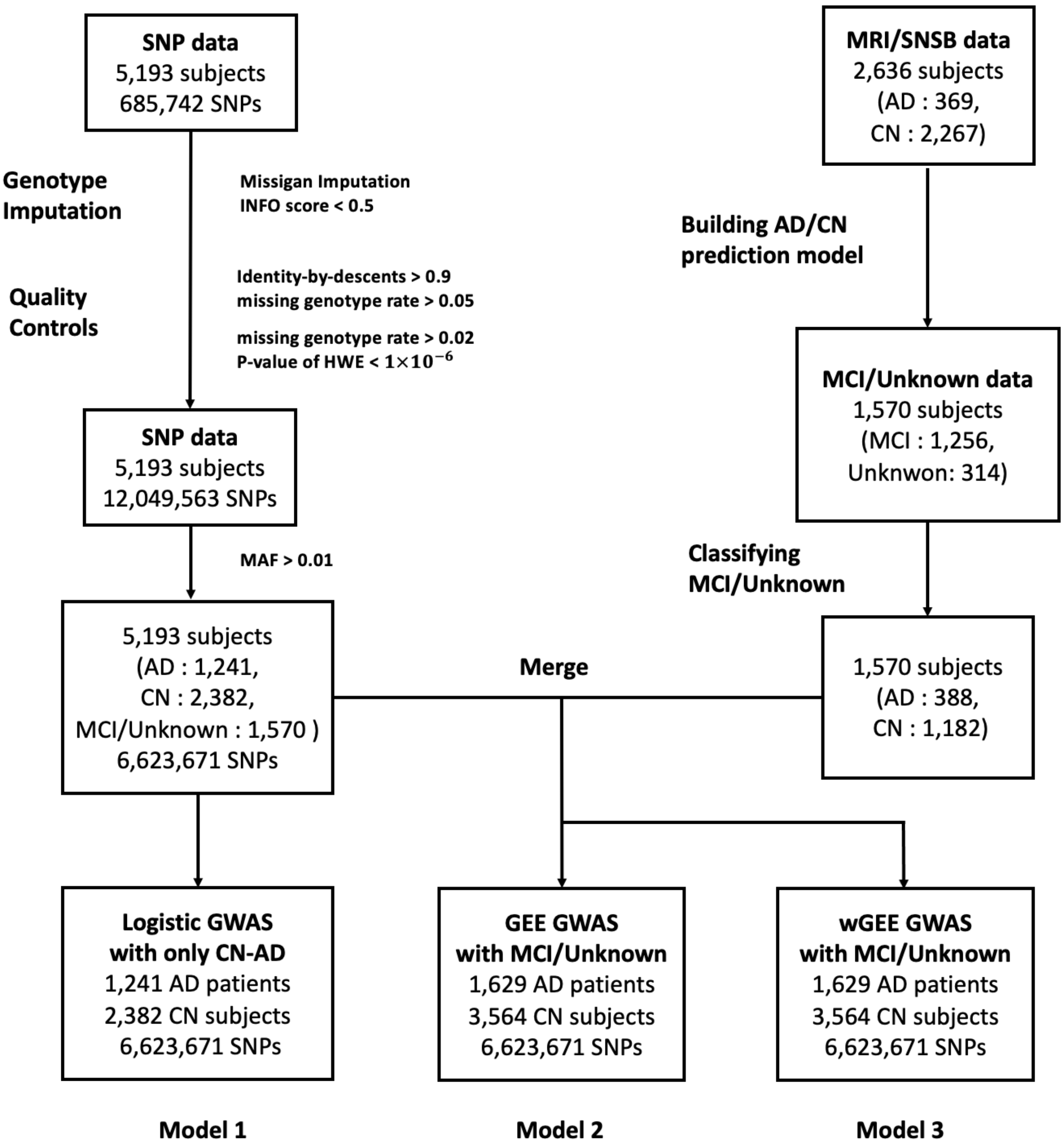

Supplementary Figure 3. Q-Q plots for genome-wide association studies using logistic regression (LR), IP, and WIP with GARD cohort.

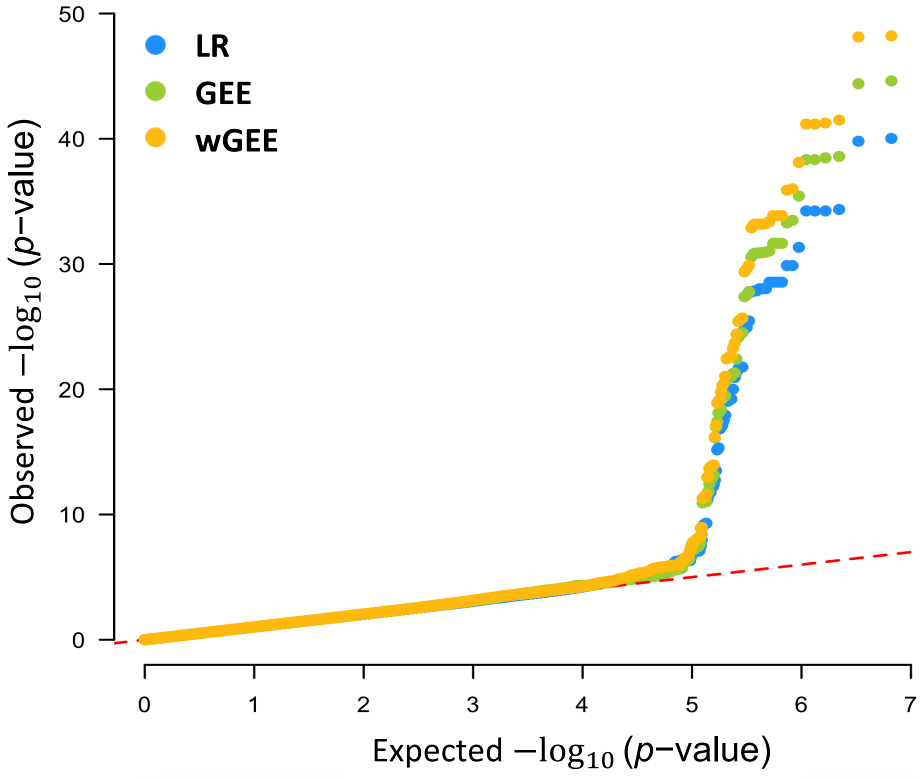

Supplementary Figure 4. Manhattan plots for genome-wide association studies with GARD cohort. (A), (B), and (C) are the Manhattan plots with WIP, IP, and LR methods. The line indicates the genome-wide significance threshold (p=5.0×10^{-8}).

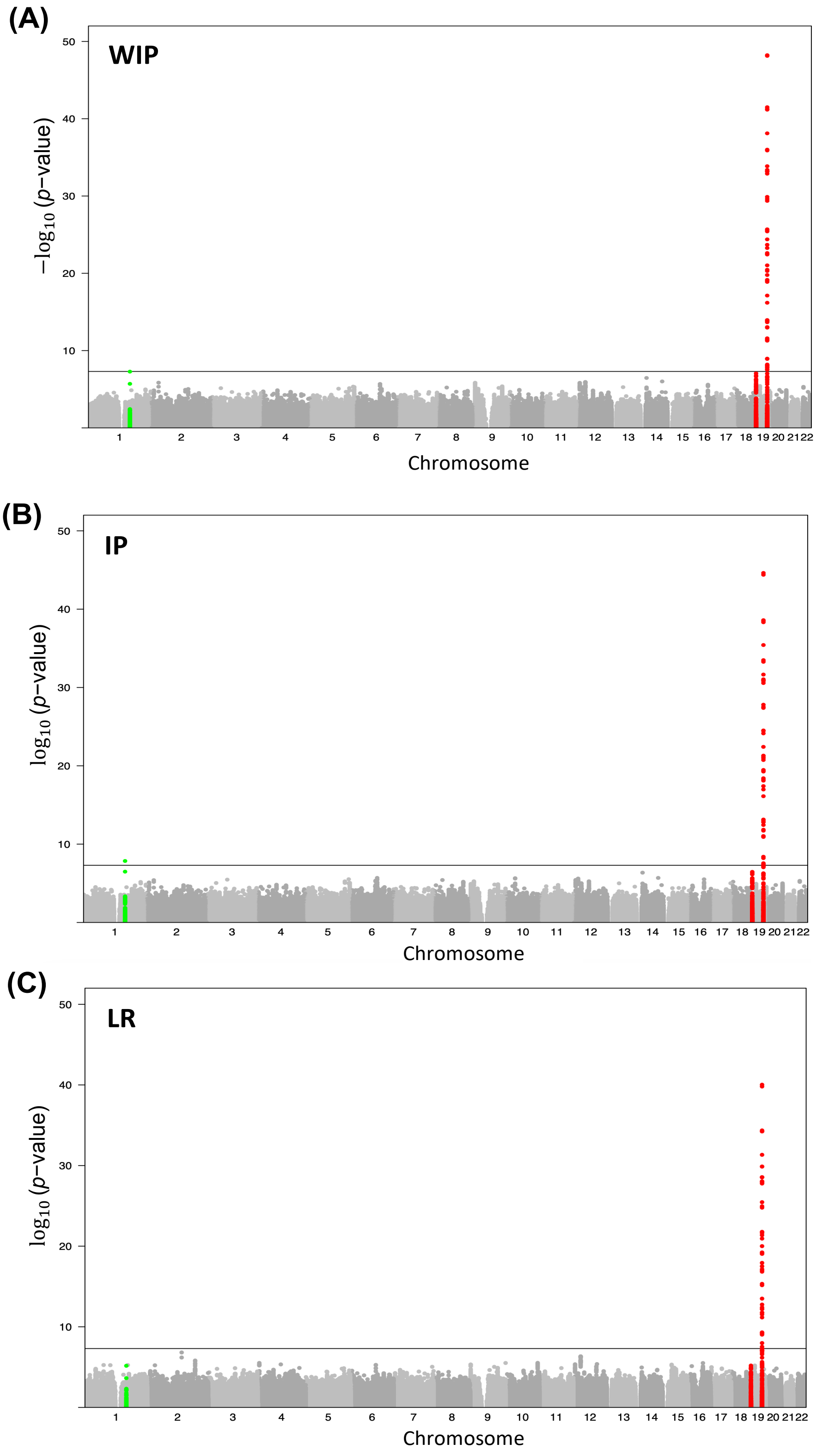

Supplementary Figure 5. Results of GWAS for GARD cohort with the weighting imputed phenotypes (WIP) method. (A) and (B) are regional plots for the associations of LMX1A and ABCA7 with AD using WIP method.

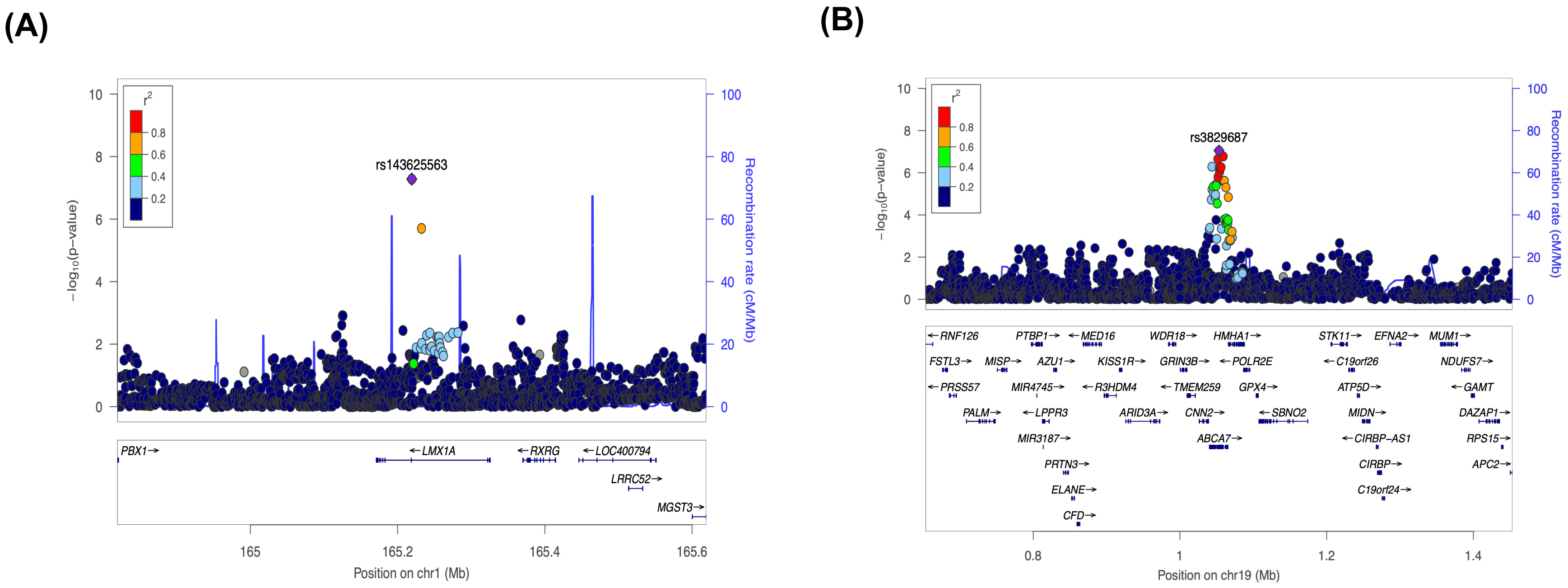

Supplementary Table 1 Characteristics of samples for prediction models.

Group	Total	CN	AD
N	2,636	2,267	369
Age, mean, years (SD)	71.9 (6.9)	71.3 (6.6)	75.3 (7.8)
Male sex, N (%)	1,114 (41.9)	928 (40.6)	186 (49.3)
Education, mean, years (SD)	10.2 (4.6)	10.5 (4.4)	8.2 (4.9)
MMSE score mean, (SD)	26.3 (3.9)	27.4 (2.1)	19.5 (5.4)
Hippocampus, mm^3 (SD)	7838 (1150)	8092 (931)	6299 (1149)
SNSB memory domain z-scores (SD)	-0.22 (1.3)	-0.05 (1.2)	-1.3 (1.5)

Supplementary Table 2 Estimated powers with simulation data. Estimated powers by logistic regression (LR) with only case/control group ($n = 10{,}000$ ($n_c = 5{,}000,\ n_a = 5{,}000$)), imputed phenotypes (IP) with GEE, and weighting imputed phenotypes (WIP) with wGEE including missing group ($n = 11{,}000, 12{,}500, 15{,}000$ ($n_c = 5{,}000,\ n_a = 5{,}000,\ n_m = 1000, 2500, 5000$)) were estimated at the nominal significant level α. K indicates the numbers of MRI traits affected by a SNP.

	**K=30**	**LR with only case/control group**			**IP including missing group**			**WIP including missing group**		
ν	$n_m(n_m/n)$	**0.05**	**0.01**	**0.001**	**0.05**	**0.01**	**0.001**	**0.05**	**0.01**	**0.001**
0.3	1000 (0.09)	0.3169 (0.0040)	0.1408 (0.0037)	0.0351 (0.0020)	0.3125 (0.0045)	0.1361 (0.0038)	0.0332 (0.0019)	0.3180 (0.0044)	0.1414 (0.0030)	0.0339 (0.0020)
	2500 (0.20)				0.3137 (0.0045)	0.1365 (0.0039)	0.0357 (0.0020)	0.3260 (0.0043)	0.1454 (0.0038)	0.0371 (0.0022)
	5000 (0.33)				0.3199 (0.0049)	0.1415 (0.0038)	0.0376 (0.0020)	0.3411 (0.0051)	0.1514 (0.0040)	0.0434 (0.0022)
0.5	1000 (0.09)	0.4537 (0.0047)	0.231 (0.0040)	0.0758 (0.0026)	0.4638 (0.0052)	0.2412 (0.0039)	0.0791 (0.0026)	0.4698 (0.0050)	0.2457 (0.0037)	0.0818 (0.0025)
	2500 (0.20)				0.4782 (0.0050)	0.2543 (0.0038)	0.0895 (0.0026)	0.4909 (0.0052)	0.2667 (0.0039)	0.0947 (0.0029)
	5000 (0.33)				0.5145 (0.0050)	0.2764 (0.0044)	0.0975 (0.0026)	0.538 (0.0049)	0.2994 (0.0046)	0.1095 (0.0027)
0.8	1000 (0.09)	0.6571 (0.0045)	0.4197 (0.0043)	0.1765 (0.0039)	0.6877 (0.0043)	0.4495 (0.0040)	0.2030 (0.0039)	0.6952 (0.0043)	0.4569 (0.0043)	0.2062 (0.0040)
	2500 (0.20)				0.7264 (0.0044)	0.4903 (0.0046)	0.2379 (0.0038)	0.7421 (0.0044)	0.5093 (0.0044)	0.2496 (0.0040)
	5000 (0.33)				0.7822 (0.0044)	0.5676 (0.0041)	0.2919 (0.0045)	0.8057 (0.0044)	0.6008 (0.0042)	0.3184 (0.0043)
1.0	1000 (0.09)	0.7814 (0.0037)	0.5603 (0.0043)	0.2897 (0.0039)	0.8116 (0.0038)	0.6101 (0.0044)	0.3251 (0.0040)	0.8173 (0.0035)	0.6168 (0.0042)	0.3325 (0.0041)
	2500 (0.20)				0.8434 (0.0035)	0.6650 (0.0045)	0.3863 (0.0046)	0.8555 (0.0036)	0.6798 (0.0042)	0.4031 (0.0047)
	5000 (0.33)				0.8975 (0.0029)	0.7429 (0.0041)	0.4872 (0.0047)	0.9126 (0.0029)	0.7668 (0.0040)	0.521 (0.0044)

Supplementary Table 3 Estimated SNP coefficients with simulation data. For positive SNP effect sizes ((β_D, β_I) = (0.049, 0.081)), SNP coefficients were estimated via logistic regression (LR) without missing group, imputed phenotypes (IP) with GEE, and weighting imputed phenotypes (WIP) with wGEE including missing group ($\hat{\beta}_{LR}$, $\hat{\beta}_{IP}$ and $\hat{\beta}_{WIP}$, respectively). We also estimated the bias between LR estimates and GEE/wGEE estimates considering the scale factor ($\hat{B}$) and direct effect ratio (d^*), so that adjusted estimates for IP and WIP ($\hat{\beta}_{IP}^{adj}$, $\hat{\beta}_{WIP}^{adj}$) were close to LR estimates ($\hat{\beta}_{LR}$).

ν	$n_m(n_m/n)$	$\hat{\beta}_{LR}$	$\hat{\beta}_{IP}$	$\hat{\beta}_{IP}/\hat{\beta}_{LR}$	$\hat{\beta}_{WIP}$	$\hat{\beta}_{WIP}/\hat{\beta}_{LR}$	d^*	$\hat{B}$	$\hat{\beta}_{IP}^{adj}$	$\hat{\beta}_{IP}^{adj}/\hat{\beta}_{LR}$	$\hat{\beta}_{WIP}^{adj}$	$\hat{\beta}_{WIP}^{adj}/\hat{\beta}_{LR}$
0	1000 (0.09)	0.046<br>(0.0005)	0.041<br>(0.0005)	90.0%<br>(2.1)	0.042<br>(0.0005)	91.3%<br>(2.0)	1.000	0.951<br>(0.002)	0.043<br>(0.0005)	94.6%<br>(2.1)	0.044<br>(0.0005)	96.0%<br>(2.1)
	2500 (0.20)		0.036<br>(0.0004)	78.0%<br>(2.3)	0.037<br>(0.0004)	80.5%<br>(2.2)		0.892<br>(0.004)	0.040<br>(0.0005)	87.4%<br>(2.3)	0.041<br>(0.0005)	90.2%<br>(2.3)
	5000 (0.33)		0.029<br>(0.0004)	63.9%<br>(2.4)	0.031<br>(0.0004)	67.5%<br>(2.3)		0.821<br>(0.007)	0.035<br>(0.0004)	77.8%<br>(2.5)	0.038<br>(0.0004)	82.2%<br>(2.4)
0.3	1000 (0.09)	0.050<br>(0.0005)	0.047<br>(0.0005)	93.9%<br>(2.1)	0.048<br>(0.0005)	95.0%<br>(2.0)	0.629	0.969<br>(0.002)	0.048<br>(0.0004)	96.9%<br>(2.4)	0.049<br>(0.0004)	98.1%<br>(2.4)
	2500 (0.20)		0.043<br>(0.0004)	86.1%<br>(2.2)	0.044<br>(0.0004)	88.4%<br>(2.2)		0.932<br>(0.004)	0.046<br>(0.0004)	92.3%<br>(2.2)	0.047<br>(0.0004)	94.8%<br>(2.2)
	5000 (0.33)		0.038<br>(0.0004)	76.4%<br>(2.4)	0.040<br>(0.0004)	79.9%<br>(2.3)		0.887<br>(0.007)	0.043<br>(0.0004)	86.1%<br>(2.4)	0.045<br>(0.0004)	90.1%<br>(2.3)
0.5	1000 (0.09)	0.053<br>(0.0005)	0.051<br>(0.0005)	95.8%<br>(2.0)	0.051<br>(0.0005)	96.8%<br>(1.9)	0.421	0.979<br>(0.002)	0.052<br>(0.0005)	97.8%<br>(2.1)	0.053<br>(0.0005)	98.8%<br>(2.0)
	2500 (0.20)		0.048<br>(0.0004)	90.7%<br>(2.3)	0.049<br>(0.0004)	92.8%<br>(2.1)		0.955<br>(0.004)	0.051<br>(0.0004)	95.0%<br>(2.3)	0.052<br>(0.0004)	97.2%<br>(2.1)
	5000 (0.33)		0.045<br>(0.0004)	83.9%<br>(2.3)	0.046<br>(0.0004)	87.3%<br>(2.3)		0.924<br>(0.007)	0.048<br>(0.0004)	90.8%<br>(2.3)	0.050<br>(0.0004)	94.4%<br>(2.3)
0.8	1000 (0.09)	0.059<br>(0.0005)	0.057<br>(0.0005)	97.8%<br>(1.9)	0.058<br>(0.0005)	98.8%<br>(1.8)	0.154	0.992<br>(0.002)	0.058<br>(0.0005)	98.6%<br>(2.0)	0.058<br>(0.0005)	99.5%<br>(2.0)
	2500 (0.20)		0.056<br>(0.0004)	95.7%<br>(2.1)	0.057<br>(0.0004)	97.8%<br>(2.0)		0.983<br>(0.004)	0.057<br>(0.0004)	97.4%<br>(2.2)	0.058<br>(0.0004)	99.4%<br>(2.1)
	5000 (0.33)		0.054<br>(0.0004)	92.2%<br>(2.2)	0.056<br>(0.0004)	95.6%<br>(2.1)		0.972<br>(0.007)	0.056<br>(0.0004)	94.8%<br>(2.3)	0.058<br>(0.0004)	98.3%<br>(2.3)
1.0	1000 (0.09)	0.061<br>(0.0005)	0.061<br>(0.0005)	99.0%<br>(1.9)	0.061<br>(0.0005)	99.9%<br>(1.8)	0.000	1.000<br>(0.002)	0.061<br>(0.0005)	99.0%<br>(1.9)	0.061<br>(0.0005)	99.9%<br>(1.8)
	2500 (0.20)		0.061<br>(0.0004)	98.7%<br>(2.0)	0.062<br>(0.0004)	100.6%<br>(2.0)		1.000<br>(0.004)	0.061<br>(0.0004)	98.7%<br>(2.0)	0.062<br>(0.0004)	100.6%<br>(2.0)
	5000 (0.33)		0.060<br>(0.0004)	98.1%<br>(2.2)	0.062<br>(0.0004)	101.5%<br>(2.1)		1.000<br>(0.007)	0.060<br>(0.0004)	98.1%<br>(2.2)	0.062<br>(0.0004)	101.5%<br>(2.1)

Supplementary Table 4 Estimated SNP coefficients with simulation data. For positive SNP effect sizes ((β_D, β_I) = (0.049, 0.081)), SNP coefficients were estimated by logistic regression (LR) with only case/control group, imputed phenotypes (IP) with GEE, and weighting imputed phenotypes (WIP) with wGEE including missing group ($\hat{\beta}_{LR}$, $\hat{\beta}_{IP}$ and $\hat{\beta}_{WIP}$, respectively). We also estimated the bias between LR estimates and GEE/wGEE estimates considering the scale factor ($\hat{B}$) and direct effect ratio (d^*), so that the adjusted estimates for IP and WIP ($\hat{\beta}_{IP}^{adj}$, $\hat{\beta}_{WIP}^{adj}$) were close to LR estimates ($\hat{\beta}_{LR}$). K indicates the numbers of MRI traits affected by a SNP (K=30).

v	$n_m (n_m/n)$	$\hat{\beta}_{LR}$	$\hat{\beta}_{IP}$	$\hat{\beta}_{IP}/\hat{\beta}_{LR}$	$\hat{\beta}_{WIP}$	$\hat{\beta}_{WIP}/\hat{\beta}_{LR}$	d^*	$\hat{B}$	$\hat{\beta}_{IP}^{adj}$	$\hat{\beta}_{IP}^{adj}/\hat{\beta}_{LR}$	$\hat{\beta}_{WIP}^{adj}$	$\hat{\beta}_{WIP}^{adj}/\hat{\beta}_{LR}$
	1000 (0.09)		0.066<br>(0.0005)	95.1%<br>(2.2)	0.067<br>(0.0005)	98.3%<br>(2.1)		0.978<br>(0.002)	0.068<br>(0.0005)	97.2%<br>(2.2)	0.068<br>(0.0005)	98.3%<br>(2.1)
0.3	2500 (0.20)	0.070<br>(0.0005)	0.062<br>(0.0005)	89.0%<br>(2.1)	0.064<br>(0.0005)	96.0%<br>(2.1)	0.452	0.951<br>(0.004)	0.065<br>(0.0005)	93.6%<br>(2.4)	0.067<br>(0.0005)	96.0%<br>(2.2)
	5000 (0.33)		0.057<br>(0.0005)	82.3%<br>(2.2)	0.060<br>(0.0005)	93.3%<br>(2.2)		0.919<br>(0.007)	0.062<br>(0.0004)	89.5%<br>(2.4)	0.065<br>(0.0004)	93.3%<br>(2.4)
	1000 (0.09)		0.084<br>(0.0005)	96.9%<br>(2.1)	0.085<br>(0.0004)	99.2%<br>(2.0)		0.987<br>(0.002)	0.085<br>(0.0005)	98.1%<br>(2.3)	0.086<br>(0.0005)	99.2%<br>(2.2)
0.5	2500 (0.20)	0.087<br>(0.0006)	0.081<br>(0.0006)	93.1%<br>(2.1)	0.083<br>(0.0005)	97.9%<br>(2.1)	0.261	0.972<br>(0.004)	0.083<br>(0.0005)	95.7%<br>(2.2)	0.085<br>(0.0005)	97.9%<br>(2.2)
	5000 (0.33)		0.077<br>(0.0005)	88.4%<br>(2.2)	0.080<br>(0.0005)	96.3%<br>(2.1)		0.953<br>(0.007)	0.080<br>(0.0005)	92.7%<br>(2.4)	0.084<br>(0.0005)	96.3%<br>(2.3)
	1000 (0.09)		0.110<br>(0.0005)	98.6%<br>(1.9)	0.111<br>(0.0005)	99.9%<br>(1.7)		0.996<br>(0.002)	0.111<br>(0.0006)	99.0%<br>(2.0)	0.112<br>(0.0005)	99.9%<br>(2.0)
0.8	2500 (0.20)	0.112<br>(0.0006)	0.108<br>(0.0006)	96.6%<br>(2.1)	0.110<br>(0.0005)	99.5%<br>(1.8)	0.081	0.991<br>(0.004)	0.109<br>(0.0005)	97.4%<br>(2.1)	0.111<br>(0.0005)	99.5%<br>(2.1)
	5000 (0.33)		0.106<br>(0.0006)	94.3%<br>(2.1)	0.109<br>(0.0005)	99.1%<br>(1.8)		0.985<br>(0.007)	0.107<br>(0.0005)	95.7%<br>(2.2)	0.111<br>(0.0005)	99.1%<br>(2.0)
	1000 (0.09)		0.128<br>(0.0005)	99.1%<br>(1.9)	0.129<br>(0.0006)	100.0%<br>(2.0)		1.000<br>(0.002)	0.128<br>(0.0005)	99.1%<br>(1.9)	0.129<br>(0.0005)	100.0%<br>(1.8)
1.0	2500 (0.20)	0.129<br>(0.0006)	0.126<br>(0.0005)	98.1%<br>(1.9)	0.129<br>(0.0006)	100.1%<br>(2.0)	0.000	1.000<br>(0.004)	0.126<br>(0.0005)	98.1%<br>(2.0)	0.129<br>(0.0005)	100.1%<br>(1.9)
	5000 (0.33)		0.125<br>(0.0005)	96.9%<br>(2.1)	0.129<br>(0.0005)	100.2%<br>(2.1)		1.000<br>(0.007)	0.125<br>(0.0005)	96.9%<br>(2.2)	0.129<br>(0.0005)	100.2%<br>(1.9)
